# Verification of the Cause of Death

**Published:** 1898-10-22

**Authors:** 


The Hospital

A Journal of

The Medical Sciences and Hospital Administration.

Edited by Sir Henry Burdett, K.C.B., and Solomon C. Smith, M.D., M.R.C.P.

Vol. XXV.—No. 630.] [Oct. 22, 1898.

### Verification of the Cause of Death.

We were once present at a London dinner party, at which one of the guests was a very pretty and charming young American widow, who, like some of her compatriots, was accustomed to display her national pride by an exaggeration of the national accent and phraseology. A metropolitan member of Parliament, whose command of aspirates was uncertain, attempted some clumsy raillery of the widow on the score of certain expressions which she thought fit to employ. She replied with almost mournful humility that she was but imperfectly acquainted with the English language, and that she had come from "the other side" mainly with the hope of acquiring mastery of it; but, she added, with a sudden and wholly unexpected feline sharpness in her voice, "guess I sha'n't take you for a professor." We were reminded of the incident by a letter which appeared in the *St. James's Gazette* of Friday from an egregious person who signed himself "Arthur Lovell," and who argued that, because the science of healing has not yet reached perfection, nay, because, in comparison with other sciences, "the supreme science of health is woefully behind," it was necessary that the work of death certification "should be constituted a special branch," or that officers should be appointed by the State, corresponding with the "mort-vérificateurs" in France, whose duty it should be to make a personal investigation of every case (of death), and who alone should be authorised to give a death certificate. A Bill for this purpose, we are further informed, has been drafted by the Association for the Prevention of Premature Burial, and in due time will be "introduced to the notice of the House of Commons." We fully admit the imperfections of the present system of death certification, but we are in no way inclined to accept Mr. Arthur Lovell as our Professor.

The object of a medical certificate of the cause—not of the mere fact—of death is twofold. In the first place, it is intended to protect the public against secret poisoning and other forms of murder. In the next place, it is intended to supply materials for trustworthy conclusions on great questions concerning the public health, such as the prevalence of preventable diseases, the influence of various conditions in increasing or diminishing the ordinary rate of mortality, and the influence of various occupations upon the longevity of those who follow them. The Legislature, by an encroachment upon the freedom of medical men which would neither have been attempted against, nor tolerated by, the members of any other profession, has had imposed upon them the duty of giving, in every case of ordinary disease which they may attend, a certificate of the cause of death for which they receive no remuneration, and which furnishes the groundwork of important national statistics. At the same time, the local registrar of deaths, often an ignorant person, is allowed to accept, in the absence of a medical certificate, any other "information" which he can obtain; information which is seldom trustworthy and frequently misleading. Cases occur in which a great infantile mortality in a particular district is not elucidated by certificates, the local registrar being content to receive "information" from old women or quacks; and it cannot be doubted that, under the facilities thus afforded, the murder of children for the sake of insurance money is a crime which, in certain districts, is largely and increasingly prevalent. The Medical Council has made repeated and strenuous efforts to diminish the evils thus arising by representing to the Government the desirableness of effective certification, based upon complete knowledge of the facts. Of course, in ninety-nine cases out of a hundred death is preceded by illness which has been under medical care and observation, and concerning which the medical attendant, who has watched the progress of the case, is able to certify without doubt or hesitation. It is in the hundredth case that the system breaks down, and the hundredth cases, added together, amount to a considerable total. For the ninety-nine any special officer is unnecessary, and all that is required is that the Legislature should so far conform to the first principles of honesty as to pay the doctor for work which they require him to perform for the advantage of the public. In the cases where doubt arises, it would certainly be useful to have special examiners, by whose reports coroners could be guided—skilled pathologists, with unrestricted power of making post-mortem examinations. As for premature burial, it is all nonsense. The supposed instances are the "gigantic gooseberries" of the French Press. The Superintendent of the Paris Morgue has for many years inquired into the authenticity of every case of the kind which has been published, and he has not been able to find a single one which had ever been heard of in the locality in which it was said to have occurred.

